# A retrospective observational study evaluating the association between vasoactive–inotropic score and mortality after major abdominal surgery

**DOI:** 10.1038/s41598-024-66641-6

**Published:** 2024-07-08

**Authors:** Jiao Huang, Jiemei Ji, Yang Zhao, Jingchen Liu

**Affiliations:** 1 Department of Anesthesiology, The First Affiliated Hospital, Guangxi Medical University, 6 Shuangyong Road, Nanning, 530021 China; 2 Department of Anesthesiology, Affiliated Hospital of North Sichuan Medical College, No.1 Maoyuan South Road, Nanchong, 637000 Sichuan China

**Keywords:** Vasoactive–inotropic score, Mortality, Abdominal surgery, Outcomes research, Medical research, Risk factors

## Abstract

The relationship between VIS_max_ and mortality in patients undergoing major abdominal surgery remains unclear. This study aims to evaluate the association between VIS_max_ and both short-term and long-term all-cause mortality in patients undergoing major abdominal surgery, VIS_max_ was calculated (VIS_max_ = dopamine dose [µg/kg/min] + dobutamine dose [µg/kg/min] + 100 × epinephrine dose [µg/kg/min] + 10 × milrinone dose [µg/kg/min] + 10,000 × vasopressin dose [units/kg/min] + 100 × norepinephrine dose [µg/kg/min]) using the maximum dosing rates of vasoactives and inotropics within the first 24 h postoperative ICU admission. The study included 512 patients first admitted to the intensive care unit (ICU) who were administered vasoactive drugs after major abdominal surgery. The data was extracted from the medical information mart in intensive care-IV database. VIS_max_ was stratified into five categories: 0–5, > 5–15, > 15–30, > 30–45, and > 45. Compared to patients with the lowest VIS_max_ (≤ 5), those with the high VIS_max_ (> 45) had an increased risk of 30-day mortality (hazard ratio [HR] 3.73, 95% CI 1.16–12.02; P = 0.03) and 1-year mortality (HR 2.76, 95% CI 1.09–6.95; P = 0.03) in fully adjusted Cox models. The ROC analysis for VIS_max_ predicting 30-day and 1-year mortality yielded AUC values of 0.69 (95% CI 0.64–0.75) and 0.67 (95% CI 0.62–0.72), respectively. In conclusion, elevated VIS_max_ within the first postoperative 24 h after ICU admission was associated with increased risks of both short-term and long-term mortality in patients undergoing major abdominal surgery.

## Introduction

Estimates suggest that over 300 million patients globally undergo surgical procedures annually, and this number continues to rise^1,2^. Despite considerable progress in surgical outcomes in recent decades, post-major surgery survival remains a significant global public health concern. This concern is particularly pronounced in patients subjected to significant abdominal surgery, placing them at a heightened risk of inadequate organ perfusion. The extended duration and intricate nature of major abdominal surgery often culminate in conditions such as hypovolemia, vasodilation, and diminished cardiac output. To counteract these challenges, the infusion of fluids and the administration of vasoactive drugs, such as dopamine, dobutamine, and norepinephrine, prove instrumental in sustaining hemodynamic stability and augmenting organ perfusion in the presence of compromised vascular tone or reduced cardiac output^3^.

The use and dosage of vasoactive drugs to sustain hemodynamics are often considered indicators of disease severity, with a prevailing belief that mortality rates increase when higher doses of these drugs are necessitated^4–6^. Vasoactive–inotropic score (VIS), first proposed in 2010^7^, was initially used to quantify hemodynamics vasoactive and inotropic support after cardiac surgery in pediatric patients, and has since been extensively used in adult cardiac surgery and critically ill patients in intensive care unit (ICU)^8–12^.VIS is a quantifying system that quantifies the amount of inotropic and vasopressor agents, including dopamine, dobutamine, epinephrine, milrinone, vasopressin, and norepinephrine^7^. Typically, it is computed as the average inotropic support administered over a predefined period. Research findings consistently assert that an elevated VIS stands as an independent risk factor for adverse clinical outcomes in various scenarios, including pediatric and adult cardiac surgery^8–10,13^, as well as in septic patients^11,12^. Koponen et al.^8^ reported that VIS_max_ provided good prediction accuracy for the adverse outcomes [area under the curve (AUC), 0.72; 95% confidence interval (CI) 0.69–0.75]. The VIS_max_ may be a useful indicator for distinguishing adult patients at high mortality risk after cardiac surgery. In patients with sepsis, VIS_max_ had a better prognostic value for 30-day mortality than (Sequential Organ Failure Assessment) SOFA score (AUC = 0.724; 95% CI 0.694–0.753)^12^, a common ICU scoring system. However, the connection between an elevated VIS and mortality in patients undergoing major abdominal surgery remains unelucidated. This study utilizes the medical information mart in intensive care-IV (MIMIC-IV) database to investigate the correlation between the maximum vasoactive-inotropic score (VIS_max_) and the prognosis of adult patients subjected to major abdominal surgery. We hypothesize that an elevated VIS_max_ within the initial postoperative 24 h is associated with both short-term and long-term all-cause mortality in adult patients undergoing major abdominal surgery.

## Methods

### Data source

We conducted a retrospective cohort study utilizing data extracted from the MIMIC-IV database^14^. MIMIC-IV version 2.2 constitutes an electronic health record dataset, which is a an extensive and publicly accessible critical care medical database. It comprised information from over 50,000 patients admitted various ICUs at the Beth Israel Deaconess Medical Center (Boston, MA, USA) between 2008 and 2019. The MIMIC project was approved by both Beth Israel Deaconess Medical Center (2001-P-001699/15) and Massachusetts institute of technology (Approval ID: 10734458). Individual patient consent was waived because unidentified health information of patients was used. A database access (certification number 1564657) was secured by one of the authors (JH). The writing of the manuscript adheres strictly to the guidelines outlined in the strengthening the reporting of observational studies in epidemiology (STROBE) statement^15^.

### Study population

We included all adult patients admitted to the ICU following major abdominal surgeries, such as colorectal, pancreatic, gastric, liver, and splenic resection. Among them, colon surgeries included left colon, right colon, transverse colon, ascending colon, descending colon, and sigmoid colon surgeries. The presence of open or endoscopic surgery, radical or palliative surgery, and oncological or non-oncological surgery were included in this study. Exclusion criteria comprised: (1) Patients lacking VIS data within 24 h post-ICU admission; (2) ICU stay duration less than 24 h. Additionally, cases with a VIS_max_ exceeding 100 were excluded, indicating an abnormal increase. In instances of multiple admissions during the study period, only records from the initial hospitalization were considered. Eligible patients were categorized into five groups, utilizing the quintiles of VIS_max_ as delineated by Koponen et al.^8^: 0–5, 6–15, 16–30, 31–45, and > 45.

### Data extraction

Navicat premium 16 was employed in conjunction with structured query language (SQL) for data extraction. The SQL script codes used herein were sourced from the GitHub repository (https://github.com/MIT-LCP/mimic-iv). Patient characteristics, encompassing age, sex, race, body mass index (BMI), and the Charlson comorbidity index (CCI), were systematically collected. Information relating to comorbidities, including hypertension, congestive heart failure, myocardial infarction, chronic pulmonary disease, cerebrovascular disease, diabetes, severe renal disease, and malignant cancer, was extracted in accordance with the international classification of diseases coding systems. Records from the initial admission to the intensive care unit (ICU) were scrutinized, encompassing the severity of illness assessed through the simplified acute physiology score II (SAPS II) and SOFA score. Relevant laboratory tests, including lactate, base excess, white blood cell count, platelet count, PaCO_2_, PO_2_, glucose level, hemoglobin level, C-reactive protein, neutrophils, bicarbonate, potassium level, calcium level, chloride level, activated partial thromboplastin time (APTT), prothrombin time (PT), and international normalized ratio (INR), were also systematically extracted. Furthermore, treatments administered within the initial 24 h post-ICU admission, such as vasopressor usage and Continuous Renal Replacement Therapy (CRRT), were documented. The VIS_max_ was computed according to the following formula, using the maximum dosing rates of vasopressors and inotropes within the initial 24 h post-ICU admission: VIS_max_ = dopamine dose (µg/kg/min) + dobutamine dose (µg/kg/min) + 100 × epinephrine dose (µg/kg/min) + 10 × milrinone dose (µg/kg/min) + 10,000 × vasopressin dose (units/kg/min) + 100 × norepinephrine dose (µg/kg/min)^7^.

### Outcomes measures

The primary outcomes of this study comprised short-term (30-day) and long-term (1-year) all-cause mortality. Secondary outcomes encompassed in-hospital mortality, as well as the lengths of both ICU stay and hospital stay. Adverse outcomes such as surgical site infections, sepsis, acute kidney injury (AKI), pneumonia, intestinal obstruction and postoperative hemorrhage within 7 days postoperatively were also extracted. We further performed subgroup analyses based on age (< 65 vs ≥ 65 yr), CCI (≤ 6 vs > 6), mechanical ventilation (yes vs no), malignant cancer patients (yes vs no), and surgery approach (open surgery vs laparoscopic surgery).

### Statistical analysis

Given the retrospective nature of this analysis, no a priori statistical analysis plan was delineated, and the sample size was derived from the available data within the database. Descriptive statistics are presented as median [inter-quartile range (IQR)] or mean (standard deviation) for continuous variables and as n (%) for categorical variables. Group comparisons of patient and clinical characteristics were conducted using the Kruskal−Wallis test for continuous variables and the chi-square test or Fisher’s exact test for categorical variables. The missing rate for each variable is outlined in Supplementary Table S1. C-reactive protein and neutrophils were eventually eliminated due to data missing exceeding 30%. Cox proportional hazard models were used to estimate the association between VIS_max_ and survival. Schoenfeld residual test and the significance of time-varying effects for each variable were used to evaluate the Cox proportional hazards model. Significance was defined by a P value of < 0.05. We performed two models to adjust for confounding factors. The confounding factors in Cox regression model were selected according to the results of univariate regression and clinical experience. Model 1 was adjusted only for age, gender, and BMI. Model 2 was further adjusted for SAPSII, SOFA, CRRT, mechanical ventilation, myocardial infarct, diabetes, malignant cancer, surgical type, surgical approach, lactate, creatinine, and INR. The cumulative incidence of 30-day and 1-year all-cause mortality in each group was analyzed using the Kaplan−Meier method, and differences were evaluated through log-rank tests. The relationships between VIS_max_ and mortality were also explored using smooth curve fitting. Logistic regression models were executed to compute the odds ratio (OR) with a 95% CI for dichotomous secondary outcomes, while linear regression was employed to assess the association between VIS_max_ and continuous secondary outcomes. Hazard ratios (HR) were calculated using the formula HR = e^βi^. The predictability of increased VIS regarding 30-day and 1-year mortality was evaluated through the area under the curve (AUC) of the receiver operating characteristics (ROC) curve. Analyses were conducted using R software (version 4.2.3; R Foundation for Statistical Computing, Vienna, Austria) and EmpowerStats (https://www.empowerstats.com) (version 4.1; X&Y Solutions, Inc., Boston, MA). A P-value < 0.05 (two-sided) was considered significant for all tests.

### Ethics approval and consent to participate

The study was approved by the institutional review boards of the Massachusetts Institute of Technology and Beth Israel Deaconess Medical Center and was granted a waiver of informed consent.

## Results

### Patient characteristics

**Figure 1:**
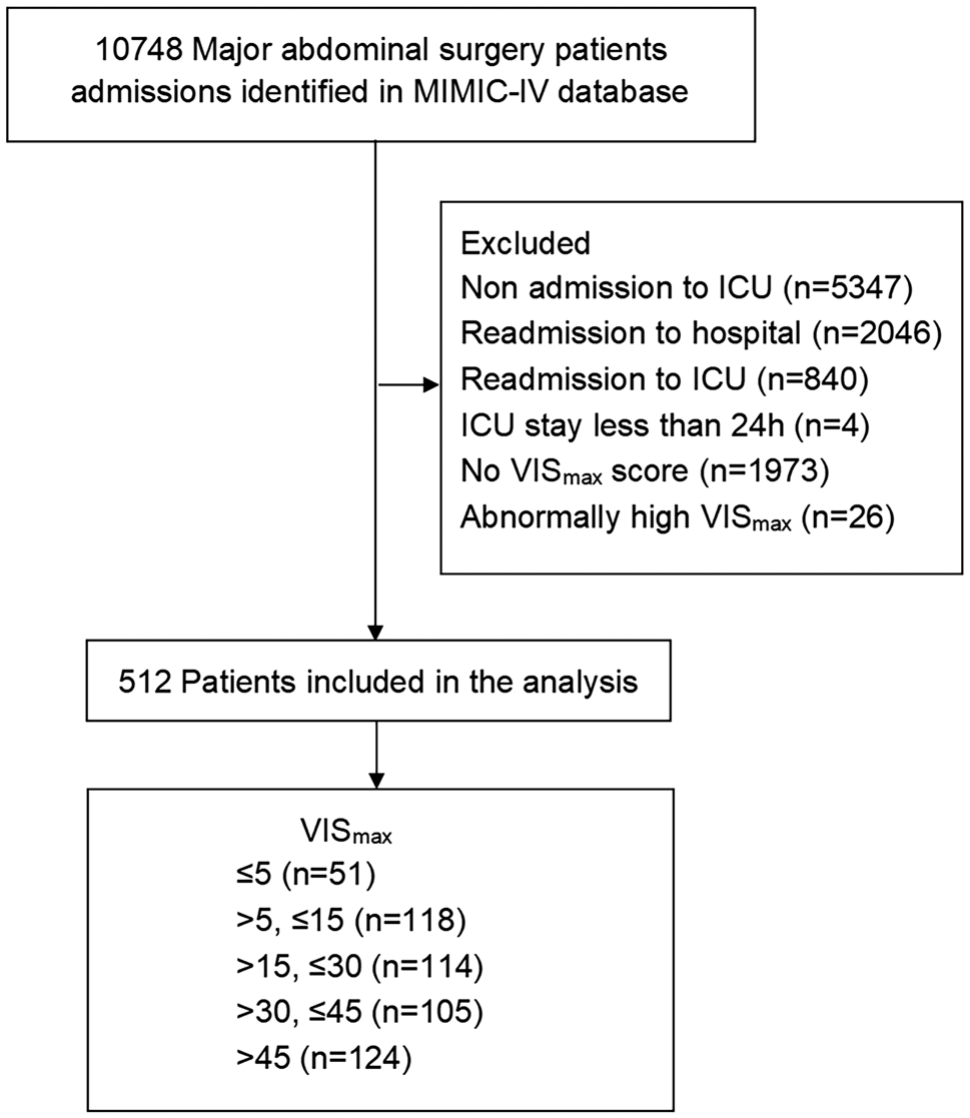
Flow chart of patient selection. MIMIC-IV, medical information mart in intensive care-IV.

Figure 1 shows the process of patient selection. Initially, 10,748 records were identified. After excluding the unqualified records, 512 patients were included in the analysis. Among them, the VIS_max_ groups were categorized as follows: ≤ 5 (n = 51), > 5−15 (n = 118), > 15−30 (n = 114), > 30−45 (n = 105), and > 45 (n = 124) (Fig. 1). Of the included 512 patients undergoing major abdominal surgery, 280 (54.7%) were male, with a median age of 67.5 years. Colorectal surgery accounted for the largest proportion among all the major abdominal surgeries included. Of 512 patients, 412 (80.5%) underwent open major abdominal surgeries. Patients who underwent open major abdominal surgery were more likely to receive vasoactive and inotropic drugs. Among the six drugs in VIS components, norepinephrine and epinephrine are the two drugs with the largest proportion. The baseline characteristics are shown in Table 1. Patients with higher VIS_max_ exhibited more severe illness (as evidenced by higher SAPS II and SOFA scores), received more treatments (such as Continuous Renal Replacement Therapy [CRRT] and mechanical ventilation), and manifested a greater prevalence of underlying comorbidities, such as diabetes. Furthermore, compared to patients with VIS_max_ ≤ 5, those with elevated VIS_max_ demonstrated higher blood lactate levels, base excess, creatinine, and poorer coagulation function. Patients with elevated VIS_max_ were also more prone to experiencing higher mortality and a prolonged ICU stay.

**Table 1:** Characteristics for patients following major abdominal surgery with different VIS_max_ score.

Variebles	VIS_max_					P-value
	≤ 5	> 5, ≤ 15	> 15, ≤ 30	> 30, ≤ 45	> 45	
No	51	118	114	105	124	
Age (yr), median (IQR)	71.6 (60.4–77.4)	66.4 (56.4–66.4)	68. (58.6–78.3)	66.5 (58.6–78.3)	68. (54.6–79.3)	0.328
Gender, n (%)						0.897
Male	29 (56.9)	66 (55.9)	62 (54.4)	53 (50.5)	70 (56.5)	
Female	22 (43.1)	52 (44.1)	52 (45.6)	52 (49.5)	54 (43.6)	
BMI (kg/m2), median (IQR)	29.5 (25.7–33.8)	29.6 (24.7–35.7)	24.5 (21.5–28.7)	27.5 (24–32.6)	27.5 (24–31.2)	0.097
Race, n (%)						0.887
White	38 (74.5)	77 (65.25)	81 (71.05)	69 (65.7)	83 (66.9)	
Other	10 (19.6)	28 (23.7)	25 (21.9)	24 (22.9)	34 (27.4)	
Black	3 (5.9)	13 (11.02)	8 (7.02)	12 (11.4)	7 (5.7)	
CCI, median (IQR)	6 (5–8.5)	6 (3.2–8)	6 (4–8)	5 ( 4–8)	5 ( 4–8)	0.195
Surgical type, n (%)						0.18
Colorectal surgery	13 (25.5)	34 (28.9)	49 (43)	37 (35.2)	46 (37.1)	
Pancreatic surgery	6 (11.8)	8 (6.8)	9 (7.9)	4 (3.8)	7 (5.6)	
Gastric surgery	11 (21.6)	15 (12.7)	9 (7.9)	13 (12.4)	17 (13.7)	
Liver surgery	7 (13.7)	18 (15.3)	18 (15.8)	12 (11.4)	9 (7.3)	
Splenic surgery	1 (2)	1 (0.8)	1 (0.9)	3 (2.9)	3 (2.4)	
Small intestine surgery	3 (5.9)	16 (13.6)	17 (14.9)	16 (15.2)	16 (12.9)	
Combined surgery	6 (11.8)	7 (5.9)	8 (7)	13 (12.4)	16 (12.9)	
Other surgery	4 (7.8)	19 (16.1)	3 (2.6)	7 (6.7)	10 (12.4)	
Surgical approach						0.2
Open surgery	41 (80.4)	86 (72.9)	95 (83.3)	86 (81.9)	102 (82.3)	
Laparoscopic surgery	10 (19.6)	32 (27.1)	19 (16.7)	19 (18.1)	22 (17.7)	
Severity of illness onset						
SOFA, median (IQR)	9 (6.5–11)	8 (6–11)	10 (8–13)	11 (8–14)	13 (11–16)	< 0.001
SAPSII, median (IQR)	42 (34.5–50)	41 (33–53)	47 (38.3–56)	50 (42–58)	60 (50–72)	< 0.001
Comorbidities, n (%)						
Hypertension	19 (37.3)	49 (41.5)	52 (45.6)	36 (34.3)	56 (45.2)	0.391
Myocardial infarct	8 (15.7)	19 (16.1)	16 (14)	10 (9.5)	11 (8.9)	0.347
Congestive heart failure	14 (27.5)	28 (23.7)	20 (17.5)	24 (22.9)	19 (15.3)	0.26
Peripheral vascular disease	6 (11.8)	17 (14.4)	23 (20.2)	20 (19.1)	23 (18.6)	0.594
Cerebrovascular disease	2 (3.9)	6 (5.1)	7 (6.1)	4 (3.8)	3 (2.4)	0.688
Chronic pulmonary disease	17 (33.3)	21 (17.8)	27 (23.7)	24 (22.9)	25 (20.2)	0.245
Diabetes	18 (35.3)	34 (28.8)	26 (22.8)	19 (18.1)	19 (15.32)	0.002
Renal disease	14 (27.5)	25 (21.2)	24 (21.1)	17 (16.2)	15 (12.1)	0.112
Malignant cancer	15 (29.4)	25 (21.2)	33 (29)	23 (21.9)	31 (25)	0.569
Severe liver disease	7 (13.7)	18 (15.3)	14 (12.3)	8 (7.6)	14 (11.3)	0.506
CRRT, n (%)	1 (2)	7 (5.9)	9 (7.90)	10 (9.5)	21 (16.9)	0.009
Mechanical ventilation, n (%)	32 (62.8)	59 (50)	78 (68.4)	82 (78.1)	100 (80.7)	< 0.001
Laboratory tests onset, median (IQR)						
Lactate (mmol L^−1^)	3.2 (1.9–4.0)	2.6 (1.8–4)	2.9( 2.1–4)	3.3 (2.1–4.4)	4.8 (3.4–7.1)	< 0.001
PO2 (mmHg)	169.7 (139.8–209)	168 (116.5–197)	169.7 (125.5–207.4)	169.7 (113.5–209.5)	168.6 (124.5–208.8)	0.482
PCO2 (mmHg)	40.7 (38– 43.3)	40.7 (36–42.5)	39 (36.5–43.4)	40.7 (36.5–45.5)	40 (34.9– 44)	0.415
Base excess	−4 (−5, −0.3)	−3.5 (−5, −1)	−4 (−6, −1)	−5 (−7, −3)	−8 (−11, −4.9)	< 0.001
Hemoglobin (g dl^−1^)	9.8 (8.7–10.9)	10.2 (8.7–11.7)	10.1 (8.8–11.2)	10.4 (9.3- 11.3)	10 (9- 11.4)	0.406
Platelets (10^9^ L^−1^)	164.3 (115.7–230.7)	183.3 (121.2–247.4)	162.3 (120.6–249.5)	183.6 (135–264)	146.4 (97.7– 244.1)	0.13
WBC (109L^−1^)	12.7 (8.6–19.5)	11.9 (8.8–16.1)	11.5 (8–16.5)	12.9 (8–17.8)	10.3 (5.9- 16)	0.068
Bicarbonate	22.5 (19.5– 24)	21 (19.1– 23)	20.8 (18.5– 23.5)	19.5 (17– 21.5)	17.5 (15–20)	< 0.001
Calcium (mmol L^−1^)	8 (7.5– 8.4)	8 (7.7– 8.5)	7.9 (7.6– 8.4)	7.9 (7.4– 8.3)	7.8 (7.4– 8.2)	0.92
Creatinine (mg dl^−1^)	1.3 (1–1.8)	1.2 (0.9–1.7)	1.3 (0.9–1.8)	1.2 (0.9–1.9)	1.6 (1.1–2.1)	0.004
Glucose (mg dl^−1^)	149 (128.5– 186.5)	135.5 (113.5– 160.1)	145.3 (114.6–169.1)	146.5 (122–178.5)	146.5 (116.8– 184.8)	0.135
Potassium (mmol L^−1^)	4.3 (3.8– 4.6)	4.2 (3.9–4.7)	4.2 (3.9–4.6)	4.4 (4–4.8)	4.3 (3.9–4.9)	0.513
INR (s)	1.4 (1.2–1.7)	1.4 (1.2–1.6)	1.5 (1.3–1.7)	1.5 (1.3–1.9)	1.7 (1.4–2)	< 0.001
PT (s)	15.7 (13.3–18.5)	15 (13.3–17.4)	16.3 (14.4–18.9)	16.3 (14–20)	18.1 (15.1–20.8)	< 0.001
APTT (s)	40 (29.3–45.6)	33 (27.9–41.4)	35.7 (30– 48.7)	35.6 (30–47)	40.7 (33.4–56.2)	< 0.001
VIS component, median (IQR)						
Dopamine	5 (3.5–5)	6 (5–10)	4 (3.5–12)	5 (5–5)	10 (7–15)	0.33
Dobutamine	5 (5–5)	3.5 (2.8–4.3)	4 (4–4)	5 (4–5)	7.6 (5.8–10)	0.22
Epinephrine	2.5 (1.8–3)	5.8 (5–6.8)	7.5 (4–17)	4 (3–5)	10.1 (6.8–12.9)	0.04
Milrinone	3.8 (3.4–4.1)	4.4 (3.1–5)	3.4 (3.2–3.6)	5 (4.4–5)	5 (4.7–5)	0.55
Vasopressin	4.1 (3.7–4.3)	4.9 (3.8–6)	5.1 (4–5.9)	5 (3.9–6.5)	5.8 (4.5–6.9)	0.1
Norepinephrine	5 (3.5–5)	10 (8–10)	20 (16–20.9)	32 (30–40)	50.1 (46.5–51.3)	< 0.001
Outcomes						
Length of hospital stay (day), median (IQR)	13.3 (9–29.6)	16 (9– 27.1)	16.9 (10.7–28)	19.8 (11.3–31.5)	17.9 (7– 32)	0.563
Length of ICU stay (day), median (IQR)	3.9 (2–7.9)	3.3 (2– 7.2)	4.9 (2.8–9.8)	5.8 (3.1–12.1)	6.8 (2.6–15.5)	< 0.001
Surgical site infections	10 (19.6)	13 (11.0)	19 (16.7)	14 (13.1)	25 (20.5)	0.262
Sepsis	19 (37.3)	51 (43.2)	61 (53.5)	69 (64.5)	81 (66.4)	< 0.001
AKI	27 (52.9)	60 (50.8)	55 (48.2)	60 (56.1)	83 (68.0)	0.023
Pneumonia	7 (13.7)	17 (14.4)	19 (16.7)	16 (15.0)	24 (19.7)	0.785
Intestinal obstruction	9 (17.6)	15 (12.7)	17 (14.9)	24 (22.4)	24 (19.7)	0.328
Postoperative hemorrhage	14 (27.5)	12 (10.2)	17 (14.9)	14 (13.1)	26 (21.3)	0.025
Hospital death, n (%)	5 (9.8)	10 (8.47)	24 (21.05)	33 (31.43)	60 (48.39)	< 0.001
30-day mortality, n (%)	5 (9.8)	12 (10.2)	23 (20.2)	31 (29.5)	53 (42.7)	< 0.001
1-year mortality, n (%)	12 (23.5)	26 (22)	48 (42.1)	53 (50.5)	74 (59.7)	< 0.001

*APTT* activated partial thromboplastin time, *BMI* body mass index, *CRRT* continuous renal replacement therapy, *CCI* charlson comorbidity index, *INR* international normalized ratio, *ICU* intensive care unit, *PT* prothrombin time, *SAPS II* simplified acute physiology score II, *SOFA* sequential organ failure assessment, *SAPS II* simplified acute physiology score II, *VIS* vasoactive–inotropic score, *WBC* white blood cell.

### Primary outcome

#### Short-term all-cause mortality (30-day mortality)

**Figure 2:**
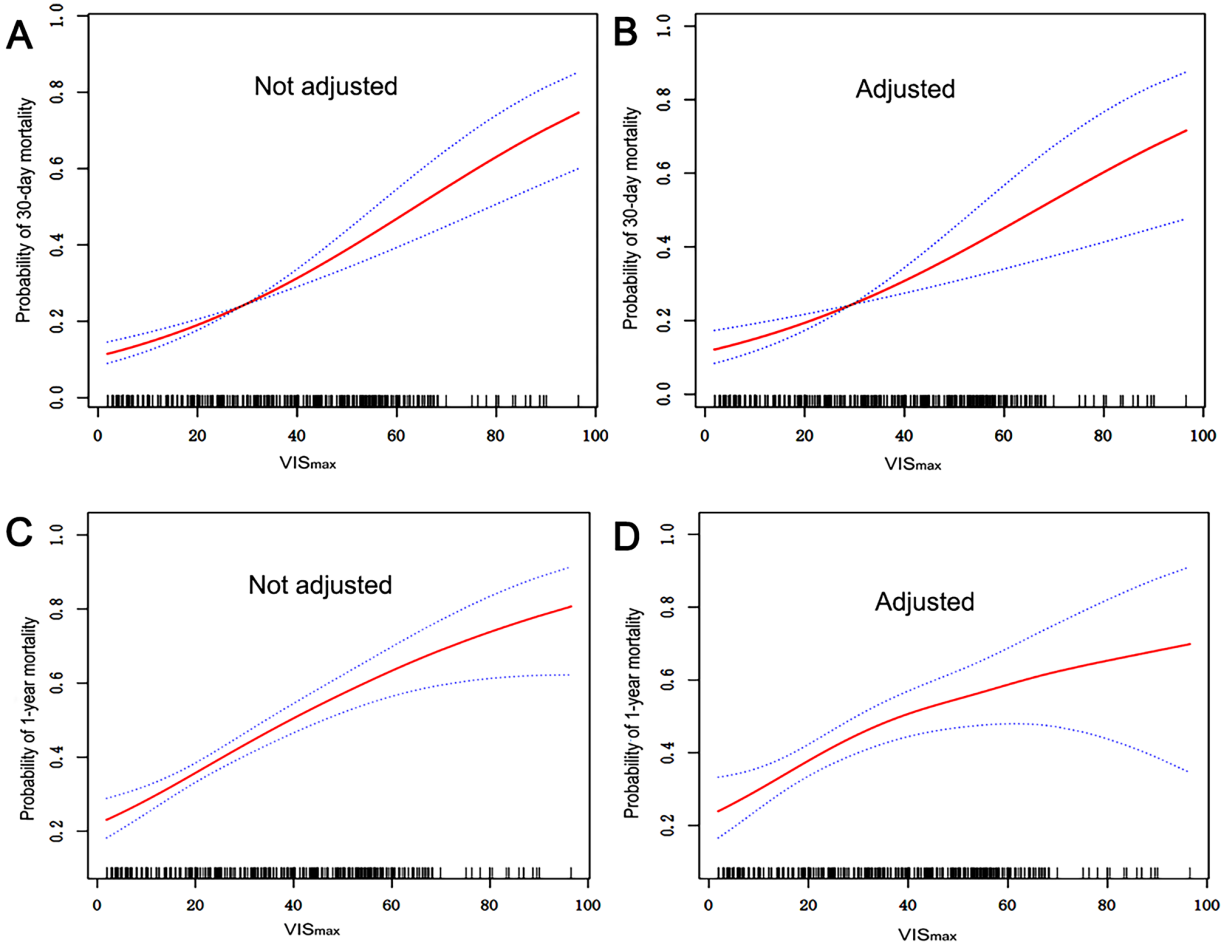
Smooth curve of the trend of the relationship between VIS_max_ and 30-day mortality (**A**,**B**) and 1-year mortality (**C**,**D**). B and D model were adjusted for SAPSII, SOFA, CRRT, mechanical ventilation, myocardial infarct, diabetes, malignant cancer, surgical type, surgical approach, lactate, creatinine, and INR.

Figure 2 illustrates the correlation between VIS_max_ and the risk of 30-day mortality. When considering VIS_max_ as a continuous variable, a notably positive association with 30-day mortality was observed in multivariable-adjusted models. This association was further evaluated through multivariable Cox regression analysis, as delineated in Table 2. In the fully adjusted model, after adjusting for SAPSII, SOFA, CRRT, mechanical ventilation, myocardial infarct, diabetes, malignant cancer, surgical type, surgical approach, lactate, creatinine, and INR. An increase of one standard deviation (SD) in VIS_max_ was linked to a 2% higher risk of 30-day mortality (HR 1.02, 95% CI 1.01, 1.03, P = 0.0037). In the evaluation of VIS_max_ on a dichotomous scale, patients in the VIS_max_ > 30−45 group and VIS_max_ > 45 group exhibited a significantly elevated risk of 30-day mortality in comparison to those in the low VIS_max_ group (≤ 5), with hazard ratios (HRs) of 3.96 (95% CI 1.26, 12.51, P = 0.02) and 3.73(95% CI 1.16, 12.02, P = 0.03), respectively (Table 2). The results of Schoenfeld residuals satisfied the proportional hazards assumption, and the DFBETA diagnostic plot indicated robust Cox analysis results (Supplementary Figure S1, S2).

**Table 2:** Association of VIS_max_ and mortality in patients after major abdominal surgery.

	Death/total, n	HR (95% CI) for mortality		
		Non-adjusted	Adjust I	Adjust II
30-day mortality				
VIS Per 1 SD		1.02 (1.01, 1.03)	1.02 (1.02, 1.03)	1.02 (1.01, 1.03)
VIS, μg·kg-1·min-1				
< = 5	5/51	1	1	1
> 5, < = 15	12/118	1.04 (0.35, 3.13)	1.22 (0.40, 3.74)	1.45 (0.42, 5.02)
> 15, < = 30	23/114	2.33 (0.83, 6.51)	2.61 (0.91, 7.46)	2.12 (0.66, 6.82)
> 30, < = 45	31/105	3.75 (1.36, 10.33)	4.65 (1.64, 13.16)	3.96 (1.26, 12.51)
> 45	53/124	7.07 (2.63, 19.02)	9.57 (3.44, 26.59)	3.73 (1.16, 12.02)
1-year mortality				
VIS Per 1 SD		1.02 (1.02, 1.03)	1.02 (1.02, 1.03)	1.02 (1.01, 1.03)
VIS, μg·kg-1·min-1				
< = 5	12/51	1	1	1
> 5, < = 15	26/118	0.92 (0.42, 2.00)	1.09 (0.49, 2.41)	1.18 (0.48, 2.92)
> 15, < = 30	48/114	2.36 (1.12, 4.99)	2.66 (1.24, 5.71)	2.40 (1.00, 5.78)
> 30, < = 45	53/105	3.19 (1.51, 6.75)	3.96 (1.82, 8.59)	3.48 (1.44, 8.42)
> 45	74/124	5.01 (2.39, 10.52)	6.37 (2.95, 13.74)	2.76 (1.09, 6.95)

Adjust I model adjust for: age, gender, BMI; Adjust II model adjust for: age, gender, BMI, SAPSII, SOFA, CRRT, mechanical ventilation, myocardial infarct, diabetes, malignant cancer, surgical type, surgical approach, lactate, creatinine, and INR.

#### Long-term all-cause mortality (1-year mortality)

In a multivariate-adjusted model, a smoothed spline demonstrates a significant positive association between increases in VIS_max_ and 1-year mortality (Fig. 2). In a multivariate Cox regression model, an SD increase in VIS_max_ was associated with a 2% higher risk of 1-year mortality in the fully adjusted model (HR 1.02, 95% CI 1.01, 1.043, P = 0.0002). When VIS_max_ was evaluated on a dichotomous scale, patients in the VIS_max_ > 30–45 group, and VIS_max_ > 45 group exhibited an increased risk of 1-year mortality, with HRs of 3.48 (95% CI 1.44, 8.42, P = 0.0055) and 2.76 (95% CI 1.09, 6.95, P = 0.0285), respectively (Table 2).

**Figure 3:**
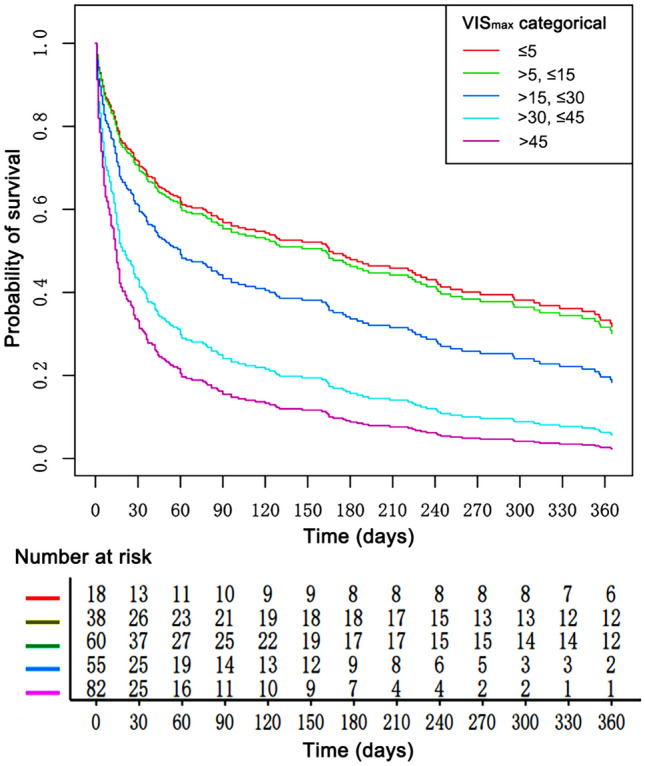
Survival curves for 5 VIS_max_ group.

Figure 3 displays Kaplan−Meier survival curves for each VIS group. The three highest VIS_max_ groups showed an increased mortality risk of up to 1 year. Compared with VIS_max_ ≤ 5 group, significant difference was observed in VIS_max_ > 30–45 group and VIS_max_ > 45 group (log-rank test P < 0.003 and P = 0.0002, respectively), but there is no difference between the two groups (log-rank test: P = 0.12) (Supplementary Table S2).

Subgroup analyses.

Table 3 shows the results of subgroup analyses for 30-day mortality and 1-year mortality. Regarding 30-day mortality, VIS_max_ was associated with an increased risk in all subgroups. For 1-year mortality, the lower limit of the 95% CI was > 1.00 in all subgroups, indicating higher 1-year mortality with elevated VIS_max_, irrespective of baseline patient characteristics.

**Table 3:** Association of VIS_max_ and 30-day and 1-year mortality in patients after major abdominal surgery.

Subgroups	No. of patients	30-day mortality		1-year mortality	
		Adjusted HR (95 CI%)	P value	Adjusted HR (95 CI%)	P value
All patients	512	1.02 (1.01, 1.03)	0.0037	1.02 (1.01, 1.03)	0.0002
Age					
< = 65	227	1.06 (1.03, 1.10)	0.0003	1.05 (1.02, 1.07)	< 0.0001
> 65	285	1.02 (1.00, 1.03)	0.0125	1.02 (1.00, 1.03)	0.00064
Charlson comorbidity index					
< = 6	302	1.02 (1.0, 1.04)	0.0252	1.02 (1.00, 1.03)	0.008
> 6	210	1.04 (1.02, 1.05)	0.0002	1.03 (1.01, 1.04)	< 0.0001
Mechanical ventilation					
No	161	1.03 (1.01, 1.05)	0.0091	1.02 (1.01, 1.04)	0.0088
Yes	351	1.01 (1.00, 1.03)	0.0268	1.02 (1.01, 1.03)	0.0003
Malignant cancer patients					
No	385	1.01 (1.01, 1.03)	0.0229	1.01 (1.00, 1.02)	0.0035
Yes	127	1.03 (1.01, 1.04)	0.001	1.04 (1.02, 1.06)	< 0.0001
Surgery type					
Open surgery	412	1.02 (1.00, 1.03)	0.0057	1.02 (1.01, 1.03)	0.0006
Laparoscopic surgery	100	1.16 (1.07, 1.26)	0.003	1.08 (1.05, 1.12)	< 0.0001

The multivariable Cox proportional hazards model was adjusted for: age, gender, BMI, SAPSII, SOFA, CRRT, mechanical ventilation, myocardial infarct, diabetes, malignant cancer, surgical type, surgical approach, lactate, creatinine, and INR.

#### ROC curve analysis

**Figure 4:**
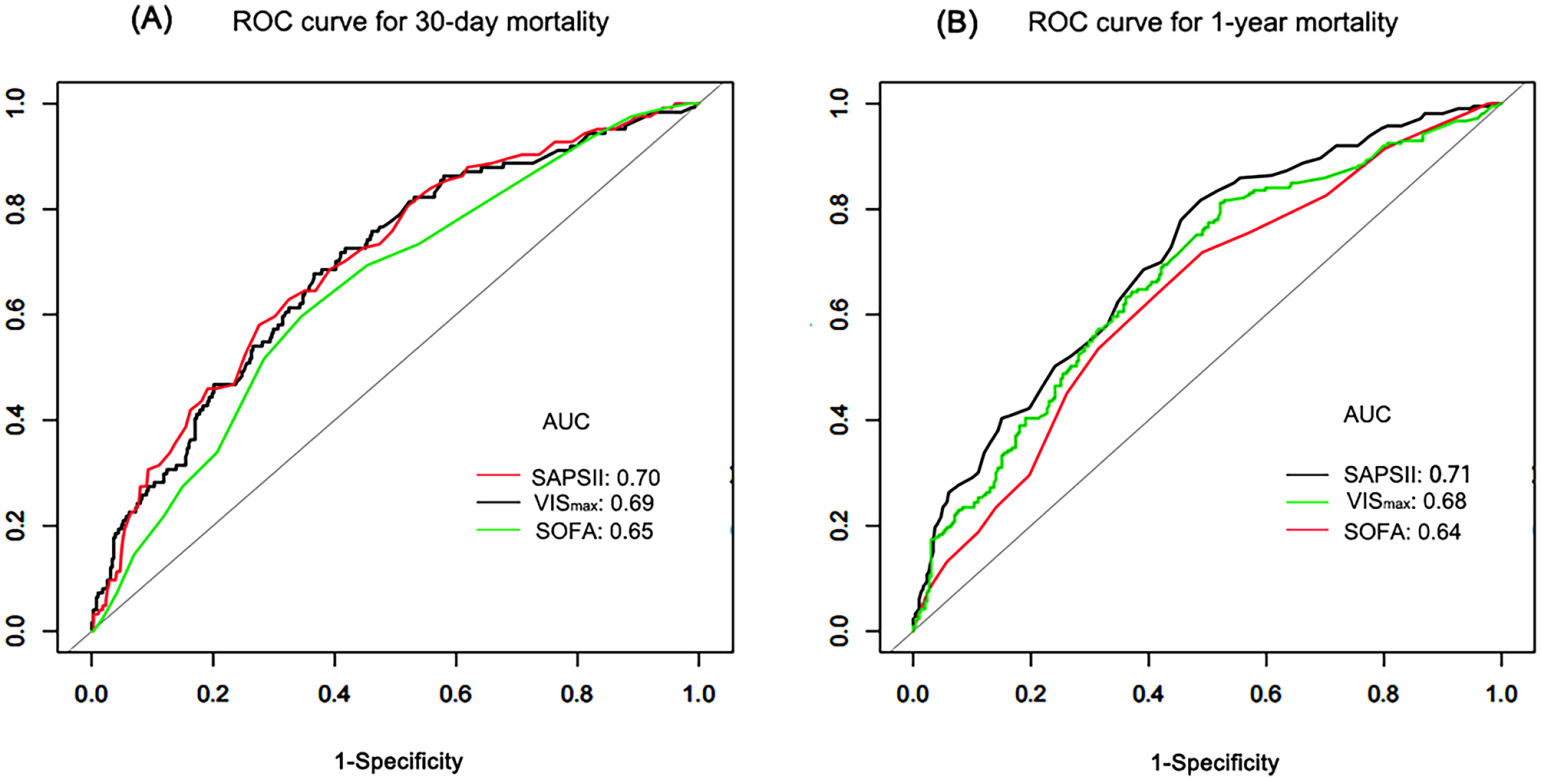
Receiver operating curves (ROC) of 30-day mortality and 1-year mortality on VIS_max_, SOFA, SAPS II. (**A**) ROC for 30-day mortality. (**B**) ROC for 1-year mortality.

VIS_max_ demonstrated a 30-day mortality prediction with an AUC of 0.69 (95% CI 0.64–0.75), exhibiting performance comparable to that of both SOFA (AUC, 0.65; 95% CI 0.60–0.69, Delong method with P = 0.13) and SAPS II (AUC, 0.65; 95% CI 0.60–0.69, Delong method with P = 0.84) (Fig. 4). This congruence in predictive capability was similarly observed for 1-year mortality. The ROC analysis for VIS_max_ predicting 1-year mortality produced an AUC of 0.67 (95% CI 0.62–0.72), comparable to SOFA (AUC, 0.64; 95% CI 0.59–0.68, DeLong method with P = 0.18) and SAPS II (AUC, 0.71; 95% CI 0.66–0.75, DeLong method with P = 0.18) (Fig. 4). The optimal cutoff value for VIS_max_ in predicting 30-day mortality and 1-year mortality was determined as 30.3 (sensitivity: 67.7%, specificity: 63.4%) and 15.9 (sensitivity: 81.2%, specificity: 41.7%), respectively.

### Secondary outcomes

#### In-hospital mortality

In contrast to the low VIS_max_ group (≤ 5), a heightened VIS_max_ was linked with a progressively elevated risk of in-hospital mortality, as revealed by the multivariable logistic regression analysis. The odds ratio (OR) for the VIS_max_ group > 30−45 was 3.59 (95% CI 1.14, 11.27, P = 0.03), while for the VIS_max_ group > 45, the OR was 3.82 (95% CI 1.19, 12.32, P = 0.02) (refer to Supplementary Table S3).

#### Length of ICU and hospital stay

The linear regression analysis indicated that an elevated VIS_max_ was correlated with a prolonged length of ICU stay, with a hazard ratio (HR) of 3.57 (95% CI 1.70, 7.37, P = 0.001). However, this association was not observed for the length of hospital stay, where the HR was 1.63 (95% CI 0.32, 8.19, P = 0.56).

#### Postoperative complications

Compared with VIS_max_ < 5 group, the elevated VIS_max_ group had a higher incidence of postoperative AKI (P = 0.023), sepsis (P < 0.001) and postoperative hemorrhage (P = 0.025).

## Discussion

To our knowledge, this is the first to study to explore the correlation between the maximum VIS and mortality in patients following major abdominal surgery. In this retrospective investigation, patients were classified into five groups according to the quintile of VIS_max_. Our findings underscore that an elevated VIS_max_ within the initial 24 h following ICU admission correlates with both short-term and long-term all-cause mortality in patients undergoing major abdominal surgery. This correlation persists across subgroup analyses. Furthermore, we noted that an elevated VIS_max_ is linked with increased in-hospital mortality and an extended length of ICU stay.

Short-term mortality rates among patients undergoing major abdominal surgery vary widely, ranging from 1 to 20%^16–19^, with higher incidence rates observed among the elderly^19^. The mean age in our study cohort was 66.2 years, and the overall 30-day mortality was recorded at 24.2%. Notably, a significant proportion of patients necessitating ICU admission following major abdominal surgery and subsequent vasoactive drug administration fall within the elderly demographic. However, our findings indicate that age does not emerge as an independent risk factor for vasoactive drug utilization. This is exemplified by our observations across the five VIS_max_ groups, where no discernible pattern has been observed associating higher scores with advanced age. The VIS, formulated through a simple formula to standardize dosages of diverse drugs and objectively quantify the degree of cardiovascular support, has witnessed growing adoption. Initially applied in pediatric and adult cardiac surgery contexts^8–10,13^, it subsequently became apparent that an elevated VIS was linked with heightened mortality in septic patients^11,12^. Our study broadens the clinical utility of VIS, illustrating that a high VIS_max_ is associated with postoperative mortality in patients undergoing major abdominal surgery. This finding aligns with earlier investigations focused on adult cardiac surgery^8–10^. A previous study indicated that patients with a high VIS_max_ level experienced prolonged hospital and ICU stays^8,10^. However, in our study, patients with higher VIS_max_ values did not exhibit a significantly extended duration of hospital stay. This discrepancy may be attributed to the increased mortality associated with elevated VIS_max_ values within the initial 24 h of ICU admission. In addition, variations in discharge criteria across different hospitals could contribute to the observed differences in length of stay.

This study has several implications for clinical practices. VIS_max_ was an independent predictor of 30-day mortality in adult patients after major abdominal surgery. It is helpful for clinicians to stratify the high risk of postoperative death in these surgical patients. In addition, VIS_max_ may be superior to traditional ICU scoring systems, such as SOFA scoring. Traditional SOFA scoring systems measure circulation dysfunction in critically ill patients and only roughly quantify the vasoactive drug support in the cardiovascular system. Given that cardiovascular dysfunction is one of the most common organ failures in the ICU^20,21^, a more elaborate assessment of circulatory function may be beneficial to improve predictive power. Several common vasopressors or inotropes used in clinical practice have also been quantified in VIS (e.g., vasopressin or dobutamine). Adding VIS to the cardiovascular component of future SOFA score may improve its circulatory dysfunction and mortality accuracy. Furthermore, considering the strong correlation between VIS_max_ and mortality, future research may consider combining VIS and other clinically relevant factors to establish a predictive model for evaluating postoperative mortality in patients undergoing major abdominal surgery, helping clinicians distinguish high-risk patients for postoperative death early and take appropriate treatment.

While the VIS is increasingly integrated into clinical practice, certain unresolved issues merit attention. Our study, akin to previous investigations by Tohmea et al.^10^, KARA et al.^22^, and Koponen et al.^8^, adopted VIS_max_ at postoperative 24 h admission to the ICU, affirming a robust association between elevated VIS_max_ and postoperative mortality. However, a study by Davidson and co-workers^23^ prospectively assessed VIS 48–72 h after infants' cardiac surgery, revealing that VIS at 48 h exhibited a more sensitive prediction of prolonged mechanical ventilation than VIS_max_ calculated within 48 h after surgery. They highlighted that the duration of vasopressor support served as a superior predictor of an unfavorable prognosis compared to the intensity of VIS. In a separate study, Crow and colleagues^24^ explored the relationship between postoperative 72 h VIS_max_ and prognosis in cardiac surgery, establishing that VIS_max_ within 48 h after surgery was correlated with prolonged ventilation time and length of hospital stay. Furthermore, the absence of a defined threshold for categorizing VIS as high or low is a current limitation. Different studies delineate patients with “high” VIS from those with "low" VIS, employing cutoff values ranging from 5.5 to 42.5^7–13,20,23,24^. Given the pivotal role of intervention timing in achieving a clinically favorable outcome, future research should prioritize determining the optimal cutoff time for designating VIS as high.

This study is beset by several limitations. First, its retrospective observational design exposes the results to potential residual bias and unmeasured confounders, notwithstanding the application of multivariable analyses. Secondly, the study did not establish a cause-and-effect relationship. Next, the patients with secondary or multiple abdominal surgeries may have a higher 30-day mortality rate, and we did not exclude this part of the patients, which may have some impact on the outcomes. Finally, the reliance of the study on data from the MIMIC-IV database, limited to United States adults, may curtail the generalizability of our findings.

## Conclusion

Elevated VIS_max_ within the initial postoperative 24 h following ICU admission was associated with both short-term and long-term mortality in patients undergoing major abdominal surgery.

### Supplementary Information

Supplementary Information.

## Data Availability

Publicly available datasets were analyzed in this study. This data can be found here:https://mimic.mit.edu/docs/gettingstarted/ .
