# Supplementary material for: A retrospective observational study evaluating the association between vasoactive–inotropic score and mortality after major abdominal surgery
Source: Sci Rep. 2024 Jul 8;14:15738. doi: 10.1038/s41598-024-66641-6 (PMC11231163; doi:10.1038/s41598-024-66641-6)
Supplement: Supplementary file 1 — Supplementary Information. [file 41598_2024_66641_MOESM1_ESM.pdf]

Supplementary Table S1

Table S1: Percentage of missing data in the variables of interest

**Variables**	**missing data (%)**
Weitht	0.59
Height	28.91
Lactate	10.16
PO2	8.20
PCO2	8.20
Base excess	8.20
Hemoglobin	0.59
Platelets	0.39
WBC	0.59
RBC	0.59
Calcium	2.15
Creatinine	0.39
Glucose	0.39
Potassium	0.39
INR	1.76
PT	1.76
PTT	2.15
Glucose	0.59
Neutrophils	33
CRP	96.7

Supplementary Figure S1

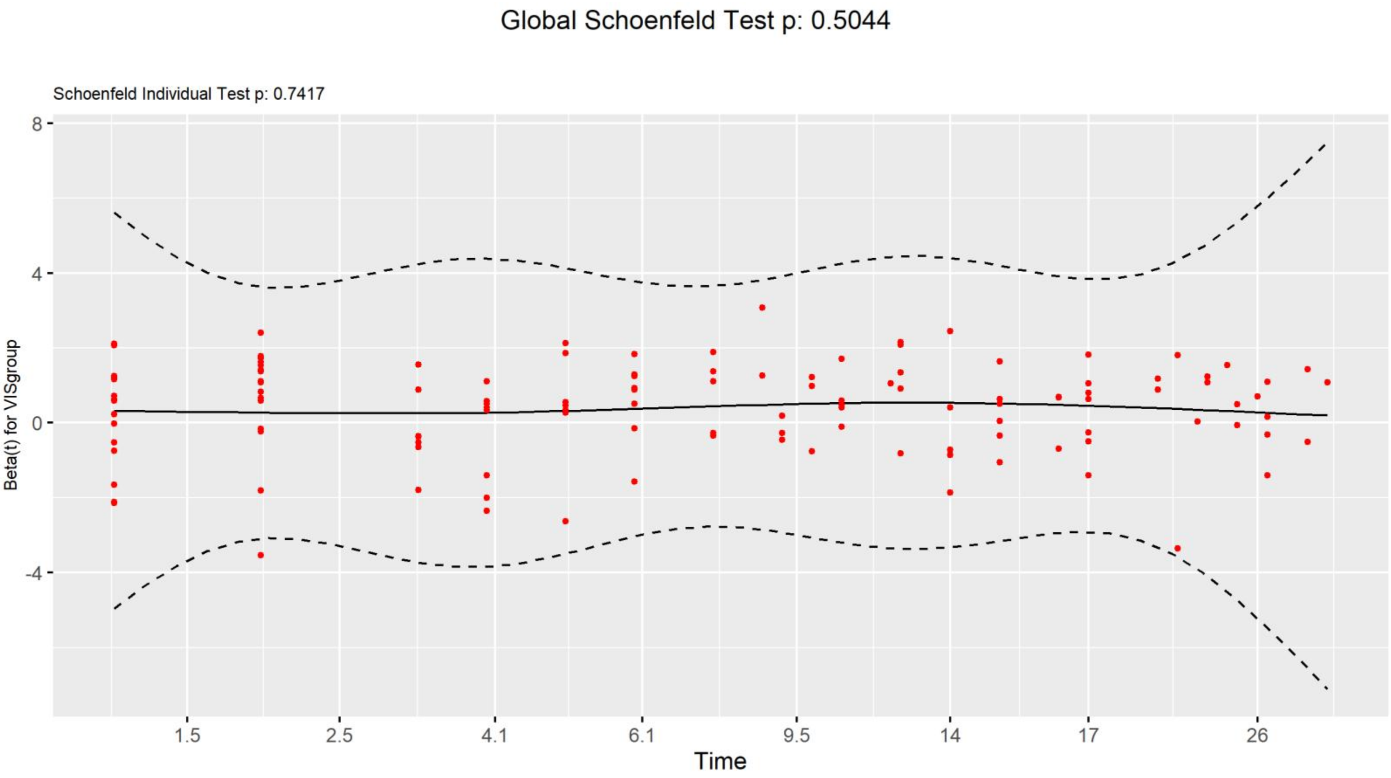

Figure S1. Schoenfeld Residuals Plot for Cox Proportional Hazards Model.

Supplementary Figure S2

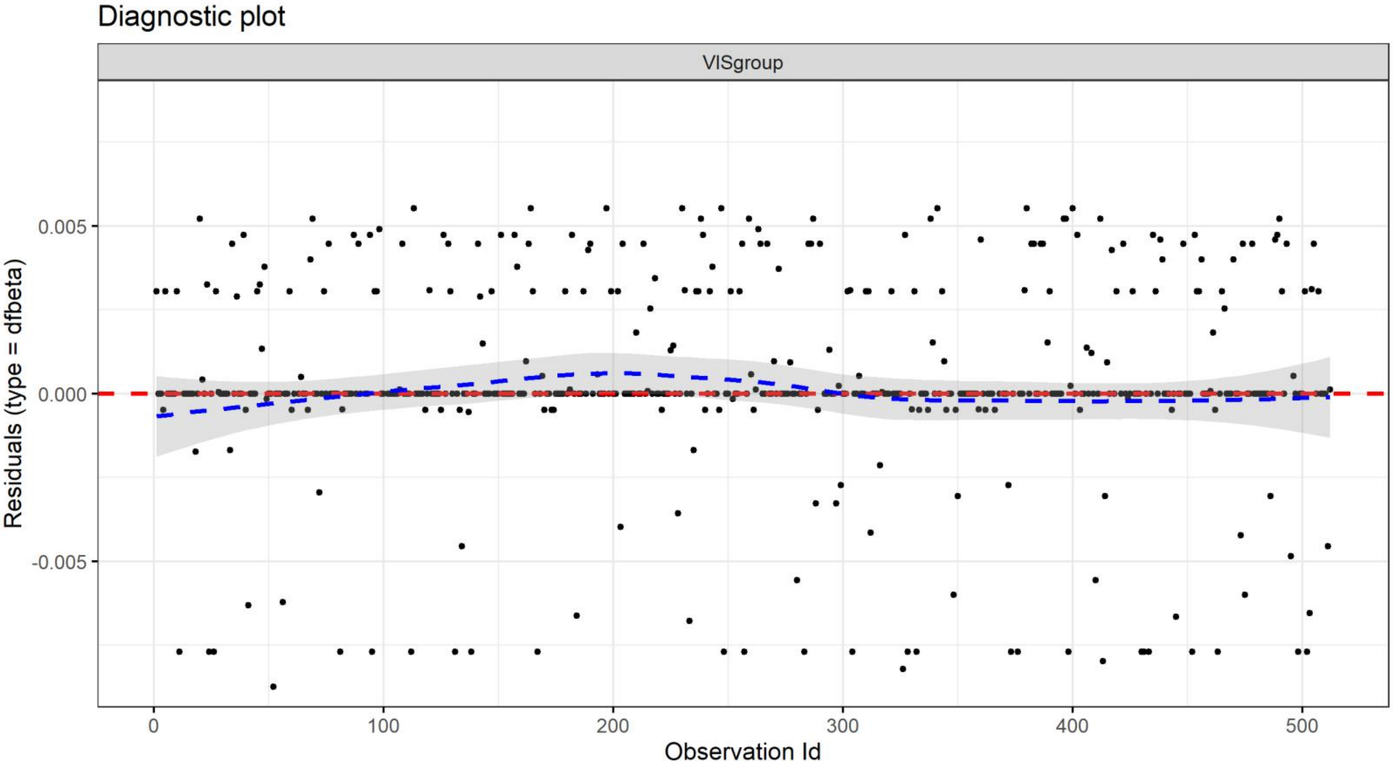

Figure S2. DFBETA Diagnostic Plot.

Supplementary Table S2

Table S2. A Log-rank test of 30-day mortality between five VIS_{max} groups.

Log-rank test	P value
Group1 and group2	0.91
Group1 and group3	0.24
Group1 and group4	0.003
Group1 and group5	0.0002
Group2 and group3	0.17
Group2 and group4	0.0001
Group2 and group5	＜0.0001
Group3 and group4	0.0072
Group3 and group5	＜0.0001
Group4 and group5	0.12

Group1: $VIS_{max} \leq 5$; Group2: VIS_{max} > 5-15; Group3: VIS_{max} >15-30 ; Group 4: VIS_{max} > 30-45; Group5: VIS_{max} > 45

Supplementary Table S3

Table S3. The association of VIS_{max} with in-hospital mortality in elderly patients after major abdominal surgery.

VIS_{max} categorical	Univariable analysis (OR, 95%CI)	P value	Multivariable analysis (OR, 95%CI)	P value
≤5	1		1	
>5, ≤15	0.85 (0.28, 2.63)	0.78	0.97 (0.27, 3.45)	0.8
>15, ≤30	2.45 (0.88, 6.85)	0.09	2.05 (0.64, 6.60)	0.15
>30, ≤45	4.22 (1.53, 11.59)	0.005	3.59 (1.14, 11.27)	0.03
>45	8.63 (3.21, 23.16)	<0.0001	3.82 (1.19, 12.32)	0.02

Adjust for: age, gender, BMI, SAPSII, SOFA, CRRT, mechanical ventilation, myocardial infarct, diabetes, malignant cancer, surgical type, surgical approach, lactate, creatinine, and INR.

STROBE Statement—checklist of items that should be included in reports of observational studies

	Item No	Recommendation
**Title and abstract**	1	(*a*) Indicate the study's design with a commonly used term in the title or the abstract
		(*b*) Provide in the abstract an informative and balanced summary of what was done and what was found
**Introduction**		
Background/rationale	2	Explain the scientific background and rationale for the investigation being reported
Objectives	3	State specific objectives, including any prespecified hypotheses
**Methods**		
Study design	4	Present key elements of study design early in the paper
Setting	4	Describe the setting, locations, and relevant dates, including periods of recruitment, exposure, follow-up, and data collection
Participants	4	(*a*) *Cohort study*—Give the eligibility criteria, and the sources and methods of selection of participants. Describe methods of follow-up<br>*Case-control study*—Give the eligibility criteria, and the sources and methods of case ascertainment and control selection. Give the rationale for the choice of cases and controls<br>*Cross-sectional study*—Give the eligibility criteria, and the sources and methods of selection of participants
		(*b*) *Cohort study*—For matched studies, give matching criteria and number of exposed and unexposed<br>*Case-control study*—For matched studies, give matching criteria and the number of controls per case
Variables	5	Clearly define all outcomes, exposures, predictors, potential confounders, and effect modifiers. Give diagnostic criteria, if applicable
Data sources/ measurement	6*	For each variable of interest, give sources of data and details of methods of assessment (measurement). Describe comparability of assessment methods if there is more than one group
Bias	6	Describe any efforts to address potential sources of bias
Study size	4	Explain how the study size was arrived at
Quantitative variables	6	Explain how quantitative variables were handled in the analyses. If applicable, describe which groupings were chosen and why
Statistical methods	6-7	(*a*) Describe all statistical methods, including those used to control for confounding
		(*b*) Describe any methods used to examine subgroups and interactions
		(*c*) Explain how missing data were addressed
		(*d*) *Cohort study*—If applicable, explain how loss to follow-up was addressed

	Case-control study—If applicable, explain how matching of cases and controls was addressed<br>*Cross-sectional study*—If applicable, describe analytical methods taking account of sampling strategy
	(*e*) Describe any sensitivity analyses

Continued on next page

**Results**		
Participants	8*	(a) Report numbers of individuals at each stage of study—eg numbers potentially eligible, examined for eligibility, confirmed eligible, included in the study, completing follow-up, and analysed
		(b) Give reasons for non-participation at each stage
		(c) Consider use of a flow diagram
Descriptive data	8*	(a) Give characteristics of study participants (eg demographic, clinical, social) and information on exposures and potential confounders
		(b) Indicate number of participants with missing data for each variable of interest
		(c) *Cohort study*—Summarise follow-up time (eg, average and total amount)
Outcome data	8*	*Cohort study*—Report numbers of outcome events or summary measures over time
		Case-control study—Report numbers in each exposure category, or summary measures of exposure
		Cross-sectional study—Report numbers of outcome events or summary measures
Main results	8-9	(*a*) Give unadjusted estimates and, if applicable, confounder-adjusted estimates and their precision (eg, 95% confidence interval). Make clear which confounders were adjusted for and why they were included
		(*b*) Report category boundaries when continuous variables were categorized
		(*c*) If relevant, consider translating estimates of relative risk into absolute risk for a meaningful time period
Other analyses	9-10	Report other analyses done—eg analyses of subgroups and interactions, and sensitivity analyses
**Discussion**		
Key results	10	Summarise key results with reference to study objectives
Limitations	13	Discuss limitations of the study, taking into account sources of potential bias or imprecision. Discuss both direction and magnitude of any potential bias
Interpretation	10-13	Give a cautious overall interpretation of results considering objectives, limitations, multiplicity of analyses, results from similar studies, and other relevant evidence
Generalisability	13	Discuss the generalisability (external validity) of the study results
**Other information**		
Funding	13	Give the source of funding and the role of the funders for the present study and, if applicable, for the original study on which the present article is based
